# Face Recognition Using Sparse Representation-Based Classification on K-Nearest Subspace

**DOI:** 10.1371/journal.pone.0059430

**Published:** 2013-03-26

**Authors:** Jian-Xun Mi, Jin-Xing Liu

**Affiliations:** 1 Bio-Computing Research Center, Shenzhen Graduate School, Harbin Institute of Technology, Shenzhen, Guangdong Province, China; 2 Key Laboratory of Network Oriented Intelligent Computation, Shenzhen, Guangdong Province, China; 3 College of Information and Communication Technology, Qufu Normal University, Rizhao, China; Dana-Farber Cancer Institute, United States of America

## Abstract

The sparse representation-based classification (SRC) has been proven to be a robust face recognition method. However, its computational complexity is very high due to solving a complex 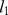-minimization problem. To improve the calculation efficiency, we propose a novel face recognition method, called sparse representation-based classification on k-nearest subspace (SRC-KNS). Our method first exploits the distance between the test image and the subspace of each individual class to determine the 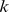 nearest subspaces and then performs SRC on the 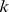 selected classes. Actually, SRC-KNS is able to reduce the scale of the sparse representation problem greatly and the computation to determine the 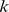 nearest subspaces is quite simple. Therefore, SRC-KNS has a much lower computational complexity than the original SRC. In order to well recognize the occluded face images, we propose the modular SRC-KNS. For this modular method, face images are partitioned into a number of blocks first and then we propose an indicator to remove the contaminated blocks and choose the 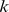 nearest subspaces. Finally, SRC is used to classify the occluded test sample in the new feature space. Compared to the approach used in the original SRC work, our modular SRC-KNS can greatly reduce the computational load. A number of face recognition experiments show that our methods have five times speed-up at least compared to the original SRC, while achieving comparable or even better recognition rates.

## Introduction

In recent years, face recognition has been, and remains being, one of the hottest and most challenging research topics in computer vision, pattern recognition and biometrics. Identifying a person by using a digital image from a photograph or a video frame sequence of him/her is the target of face recognition. There are a large number of face recognition methods, as well as their modifications. They can be categorized into two basic classes, i.e. model-based and appearance-based methods [1].

In appearance-based methods, an 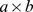 face image can be represented by a vector of 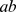-dimension. Usually, 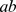 is a very big number, which leads to the dimension problem referred to as the cures of dimensionality. Therefore, a large number of linear and non-linear transform methods have been widely used at the feature extraction stage to transform images from original image space into a new low-dimensional feature space. Typical examples of linear transform methods include Principle Component Analysis (PCA) [2], [3], Linear Discriminant Analysis (LDA) [4], and Independent Component Analysis (ICA) [5]–[7]. While kernel PCA [8] and kernel LDA [9] are two widely used nonlinear transform methods.

Recently, sparse representation (SR) has shown strong ability in solving computer vision problems [10], [11]. The core issue of spare representation techniques is to solve an 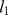-minimization problem which is equivalent to an 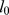-minimization problem under certain conditions [12]. Actually, the SR is based on the compressed sensing theory which is first developed in signal processing community for reconstructing a sparse signal by exploiting its sparsity structure [13]–[15]. In terms of face recognition, it is known that samples from a single class lie on a linear subspace approximately [4], [16]. Therefore, a test sample can be expressed as a linear combination of those training samples from the class to which the test sample belongs. Meanwhile, we can say that the rest training samples in the training set cannot offer a linear representation for the test sample as compact as the ones from the genuine class of this test sample. This is the discriminative nature of SRC method [12].

In theory, only a few of the computed coefficients of SRC are nonzero entries. Therefore, if there are outliers in face images, such as contingent occlusion or pixel corruptions, the representation learned by SR tends to ignore these outliers. Therefore, SRC can perform robust face recognition compared to classical methods. However, SRC has very expensive computational cost owing to the following reasons. Firstly, the sample space can be extremely large when high resolution images are used. Secondly, when seeking the sparse representation of a test sample, the whole training set is used so that the scale of the optimization could be very large. Thirdly, 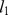-minimization algorithms used in SRC are iterative methods and the number of iterations is hard to be predicted. Although there are many 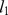-minimization algorithms, there is no clear winner that always achieves the best performance and computational efficiency [17].

As mentioned above, samples from a same class lie on the same subspace. Thus, a test sample is most likely from the subspaces which are close to it. The nearest subspace (NS) classifier is a popular and very simple method for face recognition [18] and the recently proposed linear regression-based classification (LRC) [19] can be somewhat regarded as the improvement to NS. In this paper, we propose a new scheme to perform SRC, namely SRC on k-nearest subspace (SRC-KNS), which is a two-phase method. Our method is based on the following rationale. The distance from the test sample to the individual subspace generated by a class is a measurement to indicate how well each class can represent this sample or the probability that the test sample comes from that class. That is to say, the shorter the distance that a class has, the greater the probability the test sample comes from this class. In other words, the test sample is not likely to come from the classes which have large distances. In the first phase of SRC-KNS, we compute the distances and exploit them to determine the first 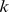 nearest subspaces for the test sample. In other words, we exploit the discriminative information from NS principle to retrieve the 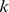 candidate classes so as to reduce the scale of the problem. The second phase of SRC-KNS performs SRC based on the training samples from the selected k subspaces. When determining the 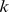 nearest subspaces, the first phase uses only the linear regression in which the *hat* matrix for each class can be calculated offline. Thus, the first phase of our method has a very low computational cost. Since we have greatly reduced the problem scale, when performing SRC, our method is computationally much more efficient than the original SRC. Moreover, the recognition accuracy of our method is comparable, even superior in some cases, to the original SRC.

In the case of dealing with the occluded face images, choosing the 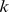 nearest subspaces usually becomes less reliable. The main reason is that the occlusion leads to biased distance computation. In this case, we propose an efficient modular approach which needs to partition each face image into a number of blocks in the same way. Since we can still assume the subspace structure for each block [19], an unconcluded block of the test sample should be well represented blockwisely by the training samples from the class of the test sample only. On the other hand, if a block is occluded, it is very likely that no class can represent it well. Therefore, for each block in a test sample, we calculate the mean distance and the minimal distance to each class and use the deviation between these two distances as an indicator to determine whether a subimage is occluded. We choose 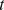 unoccluded blocks for the following operations. Then, for each class, we take the sum of distances related to the 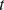 selected blocks as the final distance of this class to choose the 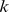 nearest subspaces. In other words, we use the 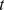 selected blocks of each images as the feature.

The remainder of this paper is organized as follows. In Section 2, we describe the proposed SRC-KNS and modular SRC-KNS. Section 3 provides the analysis and the computational complexity of our methods. In Section 4, our methods are verified by extensive experiments using standard databases. Section 5 offers our conclusion.

## Methods

In this section, we present the details of the proposed methods. First of all, we assume there are 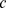 classes and 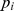 training samples in the 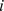
th class, 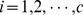. We transform all sample images into vectors 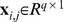, where 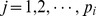 and 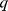 is the dimension of sample space. Then, for the 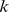
th class, we develop a class-specific dictionary 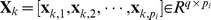, 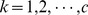 which is also known as the class-specific subspace model [16].

### SRC-KNS Algorithm

The first phase of SRC-KNS calculates the distance between a test sample 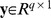 and each class-specific subspace as follows. Firstly, let 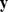 be projected onto the 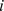 th subspace so as to obtain the predicted vector.
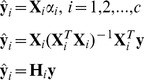
(1)where 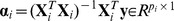 is the coding vector of the 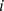
th class and 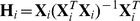 is called a *hat* matrix which maps 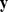 into 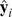. Then, the distance between 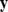 and the 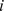
th subspace is denoted as



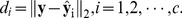
(2)We exploit 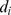 to determine the 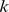 nearest subspaces that have the 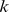 smallest distances. We refer to these 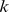 classes as the 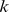 candidate classes for the test sample. The labels of these classes are denoted by 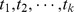, 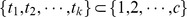.

In the second phase of SRC-KNS, we identify the test sample by SRC method using training samples from the 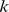 candidate classes. First of all, we need to define a new matrix 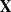 consisting of all the retained training samples:
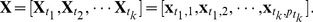
(3)


As we know, SRC tries to express the test sample as a linear combination of training samples using a coding vector 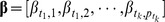. The key important feature of SRC is that it seeks a sparse solution of 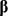 by solving a 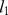-minimization problem defined as:
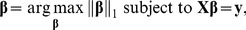
(4)or
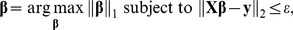
(5)where 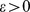 is a given error tolerance.

After introducing the two phases of the proposed method, we summarize the main steps of SRC-KNS as follows:

Step 1. Choose 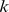 candidate classes according to (1) and (2);

Step 2. Solve the optimization problem (4) or (5) to find the coding vector 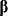 by using the training sample from the 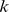 candidate classes;

Step 3. Calculate the residuals 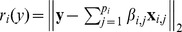, 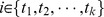;

Step 4. Classify the test sample into the class that has the minimum residual 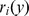.

### Decision of the K Nearest Subspaces Using Modular Scheme

In some real-world face recognition scenarios, face images could be partially occluded. Usually, an occlusion covering on some patches of a test image can be assumed to be local and connected. In the sample space, a contaminated test image can be far away from its unoccluded counterpart. Thus, the distance between the occluded image and each class-specific subspace becomes unpredictable, which makes NS method difficult to retrieve candidate classes correctly [16]. It is well known that using the modular representation approach is helpful to deal with partially occluded case [20]. Thus, we partition the images into a number of blocks and process each block independently. For a block, if we can find out whether it is occluded, using those unoccluded blocks to recognize the subject should improve the accuracy of a classifier. In the next paragraph, we describe the proposed modular SRC-SNS and the detailed explanation of the motivation of our modular scheme is given in the next section.

For each block of a test image, the 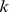 nearest subspaces of it can be determined using the distance as SRC-KNS, but blockwisely. However, it is not necessary that every block determinates the same 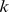 nearest subspaces. Particularly, the blocks contaminated by occlusion are not useful to make any decision. In this paper we propose a modular scheme to decide the 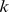 nearest subspaces. The core idea of our scheme is that we first identify the unoccluded blocks of a test sample and use them as new feature to do classification.

Let us suppose that training images are partitioned into 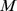 blocks and each block is converted into a vector 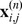, (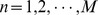, 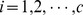, and 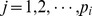). The dictionary of the 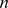
th block of the 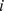
th class (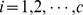) is defined as:
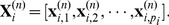
(6)


We also partition a test sample into the same 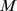 blocks and each block is denoted as 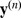 (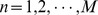). The distance between 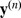 and its projection on the 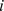
th class is computed
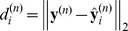


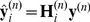
(7)

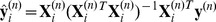
where 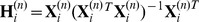.

Among the 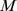 blocks of the test sample, the unoccluded ones can be approximately represented by the corresponding blocks of the training samples from the class of the test sample. However, one cannot expect any class is capable of providing a valid representation of the occluded blocks in the test sample. Further, the occluded block locates very far away from all blockwise subspaces 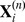 and the distances between the occluded block and blockwise subspaces does not vary too much. That is to say, for an occluded block, there is little difference among all 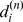
(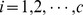). To formulate our idea, we use the following variable as an indicator to identify unoccluded blocks:
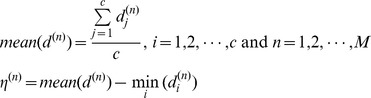
(8)where 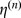 is used to identify whether the 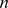
th block of a test sample is occluded. Then, by using this indicator, we choose 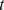 blocks which have the first 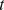 largest values of 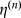 as unoccluded blocks. We denote the selected blocks as 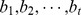, so that 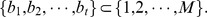


After identifying unoccluded blocks, we use these blocks to form the new feature for all samples and redefine the distance between 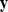 and each class as:
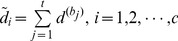
(9)


In this way, the new distance measurement can avoid the biased estimation caused by occlusion. We take the 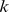 subspaces that have the 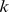 smallest 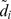 as the nearest subspaces for the test sample. Since the blocks with occlusion are not useful for classification, we define a new feature space for each class only using the clear blocks as.
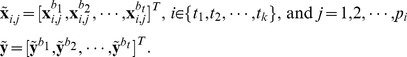
(10)


Finally, we perform SRC using the new feature space on all the samples from the selected classes. The detailed classification steps of our modular approach are summarized as follows:

Step 1. Divide the samples into 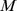 blocks and compute 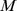 distances 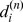 for each class;

Step 2. Calculate the indicator 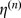 of each block to identify the unoccluded blocks;

Step 3. Determinate the 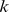 nearest subspaces;

Step 4. Perform SRC on the training samples from the selected classes in thenew feature space.

## Analysis

In this section we analyze the justification and rationale of our method.

### Motivation of SRC-KNS

Face images from the same class tend to exhibit a degenerate structure, though they are in a high dimensional image space. That is to say they lie on, or nearly, a same subspace. Based on this concept, the nearest subspace classification(NSC)was proposed to recognize face images [18], [19]. For NSC, the discriminative information is provided by measuring the distances between a test sample and all class-specific subspaces. However, some literatures suggested that using the global representation scheme can achieve higher recognition accuracy than NSC [21]. That is, a test sample is represented by the training samples across multiple classes. For example, SRC uses global scheme to choose the minimal number of training samples to represent the test sample. However, to solve the 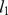-minimization problem in SRC has a heavy computational load. In [17], some fast 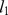-minimization algorithms are compared, but no clear winner that always achieves the best performance and efficiency is found for face recognition. It is always interesting to find new methods which improve the computation efficiency without decrease of the recognition accuracy. Commonly, a variety of factors determine the speed of an SRC algorithm, such as the dimension of the sample space, the size of the dictionary, and the internal structure of subspaces.

Although NSC cannot outperform SRC [12] in general, the 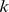 selected classes retrieved by using the distance between the test sample and each class-specific subspace can be very likely to include the genuine class of a test sample. Because it is unlikely that the training samples from classes whose subspaces are very far from the test sample can represent it better than ones associated with the near subspaces. Therefore, for SRC, the training samples from the selected classes can accomplish this representation more promisingly than those associated with the far subspaces.

**Figure 1:**
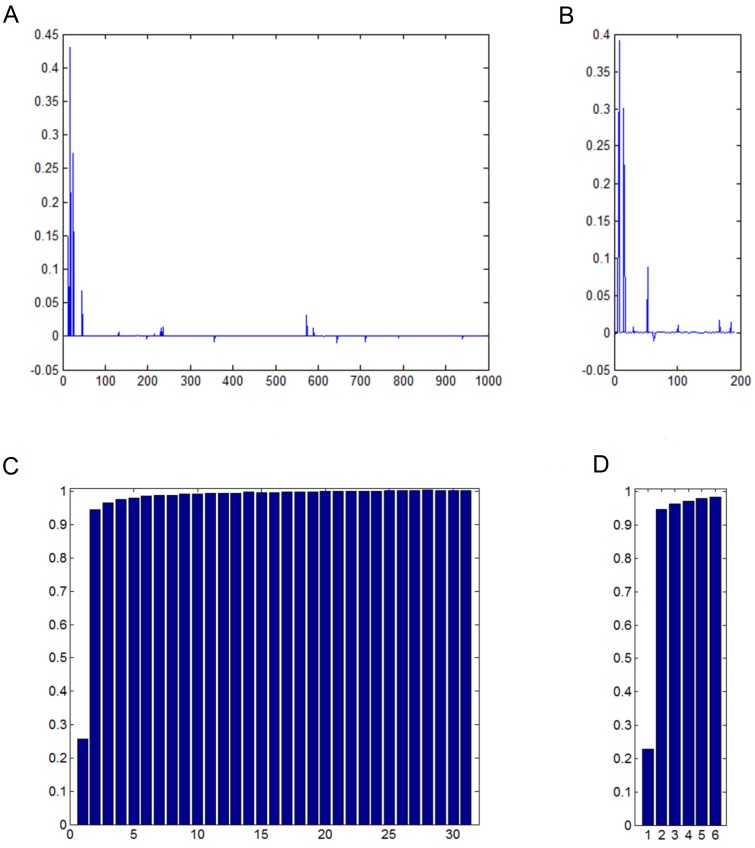
Example of the sparse representation of a test sample (31 subjects). (A) Sparse coefficients for SRC using the entire training set. The training samples are arranged in the order of the distance of their class to the test sample (from the left, the class has the smallest distance). (B) Sparse coefficients for our SCR-KNS (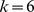). (C) The mean residuals in the case of using the training samples from all classes. Each time the residuals are computed, the values are arranged in the order of the distance. (D) The average residuals for SCR-KNS (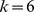). Note that the smallest residual is slightly smaller than that in (D).

By showing a demonstration in Fig. 1, we want to put forward the following points. Firstly, the classes having the large distances are not likely to be the one which the test sample is from. As shown in Fig. 1 (A), large coefficients only tend to distribute on training samples from the classes whose subspaces are close to the test sample. Secondly, after cutting the classes associated with the subspaces which have big distance to the test sample, the sparse nature of the coding coefficients is unchanged because the genuine class of the test sample is usually contained in the selected classes, as shown in Fig. 1 (A) and (B). Thirdly, the 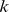 classes selected by NS contain the class of the test sample with very high probability. This point will be demonstrated in the experiments. Fourthly, the class of the test sample becomes more significant in representing the test sample after reducing the size of the training sample set, which can be seen by comparing Fig. 1 (C) with Fig. 1 (D). This might increase the recognition accuracy.

### Explanation for the Modular Approach

Under occlusion conditions, a part of the pixels, known to be connected, in a test sample is contaminated. We cannot reliably show the genuine relationship between the test sample and each class by the distance measurement, especially when the contaminated portion is large. Therefore, we propose a modular approach to address this problem. This approach has two roles, i.e. to identify the uncontaminated blocks and to choose the 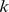 nearest subspaces.

**Figure 2:**
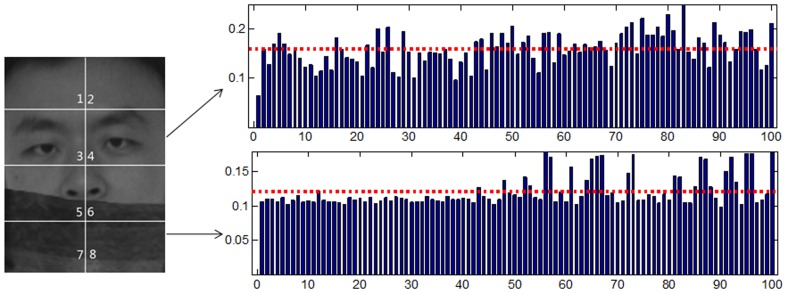
Demonstration of the idea of the modular SRC-KNS. The test sample (from class 1) with scarf occlusion is divided into 8 blocks. The distances 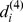 of the fourth block are shown in the top right while the distances of the eighth block 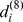 are shown in the bottom right. The dotted lines indicate the mean distance for that block. 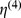 is significantly bigger than 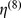. Therefore, using blocks with bigger 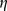 can correctly identify the test sample, while the blocks with smaller 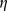 are not helpful for classification in this case. The subject of the photograph has given written informed consent, as outlined in the PLOS consent form, to publication of their photograph.

In the modular approach, images are represented by 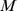 blocks and we compute the distances for each block independently. We still assume that each block from a same class lie on a linear subspace. Thereby, we have two important inferences. Firstly, a block of the test sample can be represented approximately by corresponding blocks of the training samples from the class of that test sample, whereas other classes can only provide poor representations. Secondly, if blocks are occluded, no class can represent the blocks much better than the others (see Fig. 2). Inspired by these two points, we consider to use the distribution of the distances 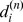 to distinguish whether a block is occluded or not. Our scheme is to use how much the minimal distance deviates from the mean distance as an indicator, i.e. 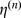, to decide whether a block is occluded, as shown by (8). Big 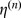 means one distance deviates the average greatly, which means the corresponding block is very likely to be clear since one class can express this block well. On the other hand, small 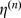 means distances are close and no class can express the test sample better than others, which implies this block is occluded.

Moreover, for the partially occluded blocks, the contaminated part decreases the representation accuracy of the correct class. That is to say, those blocks have smaller 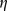 than the uncontaminated ones but bigger 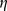 than the occluded ones.

### Computational Complexity

Solving the 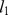-minimization problem in SRC is the core of finding the sparse coding vector. The classical solution to 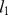-minimization, as presented in (4), has a computational cost of 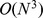 [22] in each iteration, where 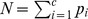 which is the number of the training samples, i.e. used in SRC,. And it requires 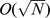 iterations in total. Thus, the computational cost is very expensive for many real-world applications. In the past few years, many fast 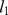-minimization solvers of higher efficiency have been developed [23]–[27]. Generally, for a 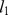-minimization solver, besides the dimension of the feature space, the size of dictionary and the number of nonzero entries in the coding coefficients are two main factors deciding the computational cost. For example, homotopy 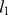-minimization [24] solver can find solution using only 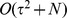, where 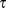 is the number of nonzero coefficients. It has been shown that using the extreme sparse coding vector cannot produce good results for face recognition problem [21]. Thereby, 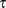 could not be very small in common. Moreover, 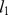-minimization solvers are iterative algorithms and the number of iteration steps are hard to predict for some algorithms. Actually, it is difficult to assess the exact computational cost for face recognition applications. But the size of the training set used in SRC does have great effect on speed.

In SRC-KNS, the scale of the 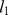-minimization problem becomes much smaller than the original SRC since we can set 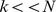. However, we still need to analysis the computational complexity for the first phase which is an extra procedure for SRC. Note that when performing face recognition, all hat matrices 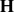 can be computed before recognition. We can regard this procedure as model training which has computational cost of 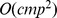 (Here we assume each class has 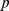 training samples). When recognizing faces, we need to calculate some matrix multiplications and distances in the first phase which needs computational cost of 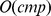 which is much smaller than that of SRC. Note that our modular approach has the same computational cost as SRC-KNS because no extra operation is required except partitioning the images.

In summary, compared to the original SRC, the additional procedure added to find the 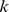 candidate classes in SRC-KNS has quite low computational complexity. But it can significantly reduce the scale of the 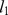-minimization problem solved in performing SRC. Therefore, the total computational cost of SRC-KNS decreases, which is verified in the experimental results.

## Experimental Results

In this section, we verified the effectiveness of our methods by performing experiments on publicly available databases. Mainly, we want to examine how 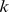 affects computation time and recognition accuracy. First, we tested SRC-KNS on three databases with different scales under normal conditions to verify the efficiency despite the variation of database size. Then, we tested how random pixel corruption influences the results. Finally, our modular approach was tested using face images with sunglasses and scarf occlusions. All experiments were implemented using the Matlab on a PC with an Intel i7-2600K Processor.

As shown in [12], [19], to recognize facial images in higher feature space can obtain better identification effect. In our experiments, we used images whose features are more than the size of training set. Hence, a test sample can only be approximately expressed by the training samples from the genuine class, so that 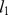-minimization problem solved here is formulated as (5). An 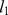-minimization algorithm, call L1LS, was used to solve the 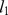-minimization problem The L1LS has been shown high recognition rate and good computational efficiency for face recognition [17]; it recasts the problem of (5) as LASSO problem with a Lagrangian multiplier 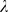. The Matlab toolbox of the L1LS is available at [28]. We set 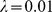 in all experiments.

### Test SRC-KNS Method on Different Size Databases

The previous studies showed that the downsampled face images can serve as face feature very well. In the experiments, we downsampled the original images to a moderate dimension. We compare SRC-KNS with the original SRC and the latest NSC, namely LRC.

#### Extended Yale B database

**Figure 3:**
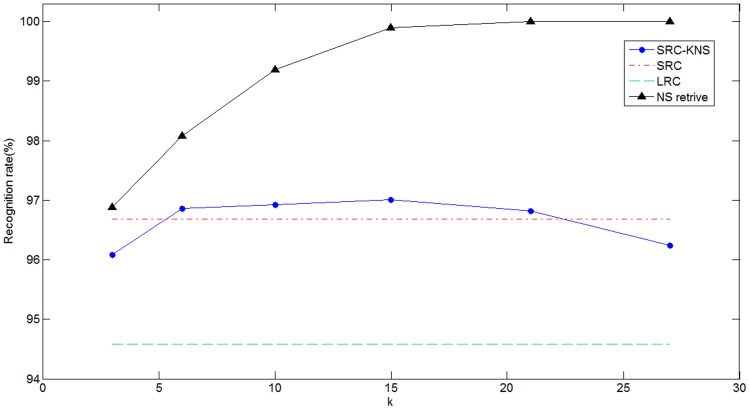
Recognition accuracy on the Extended Yale B database.

The Extended Yale B database includes 2,414 face images of 38 subjects captured under various lighting conditions [29]. We only used a subset of the original dataset consisting of 31 subjects each of which has 64 images (because some images of the other 7 unused subjects were damaged). For each subject, 32 images were randomly selected to serve as training samples and the rest were used for testing. We repeated 10 times so as to show the mean results, which avoids the special choice of the training data. We downsampled the original images to the size of 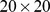. Our SRC-KNS was tested using 6 different 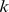
(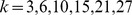). The mean computation times of recognizing a test sample for SRC-KNS and SRC are compared in Table 1. Meanwhile, we illustrate the recognition rates of SRC-KNS, SRC, and LRC in Fig. 3. Two SRC-based methods attained similar recognition rates which are higher than that of LRC. The SRC-KNS is significant faster than SRC which achieves about 5 times speed-up when 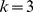. We also illustrated the retrieval accuracy of NS in fig. 3. The retrieval rate increased with 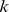 and approached 100% when 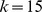. We can see the retrieval rate of NS is higher than recognition rate of SRC, which insures that SRC-KNS has comparable recognition accuracy as the original SRC.

**Table 1:** Comparison of mean CPU time(measured in second) on the Extended Yale B database.

Method	SRC-KNS						SRC
	*k* = 3	*k* = 6	*k* = 10	*k* = 15	*k* = 21	*k* = 27	
CPU time	0.508	0.731	0.993	1.420	1.727	2.058	2.503

#### AR database

**Figure 4:**
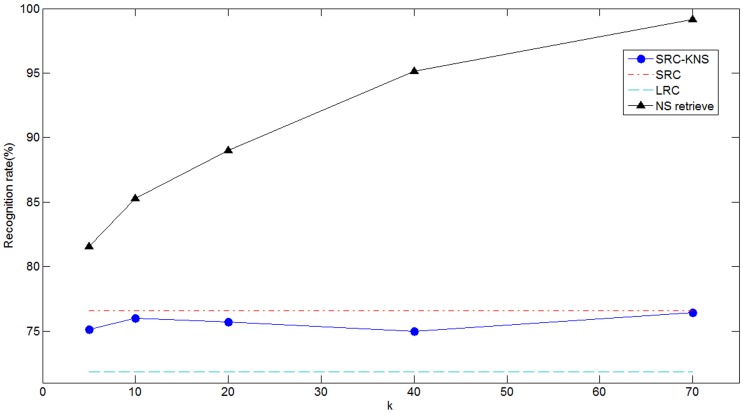
Recognition accuracy on the AR database.

There are 126 subjects in the AR database [30]. For each subject, 13 images were captured in the first session, and the other 13 images were taken in the second session. In this experiment, we use a subset of 100 individuals from the AR database consisting of 50 male subjects and 50 female subjects. And 7 images without any occlusion from Session 1 were used for training; the corresponding 7 images from Session 2 were used for testing. All used images were downsampled to 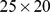. We tested SRC-KNS with 5 different 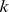 (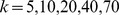). The results including CPU time and recognition rates are shown in Table 2 and Fig. 4 respectively. The recognition rates of our method are comparable to SRC and 3% higher than that of LRC at least. When using 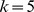 our method can obtain around 10 times speed-up compared to SRC.

**Table 2:** Comparison of CPU time on the AR database.

Method	SRC-KNS					SRC
	*k* = 5	*k* = 10	*k* = 20	*k* = 40	*k* = 70	
CPU time	0.499	0.645	0.868	1.589	3.074	5.174

#### FERET database

**Figure 5:**
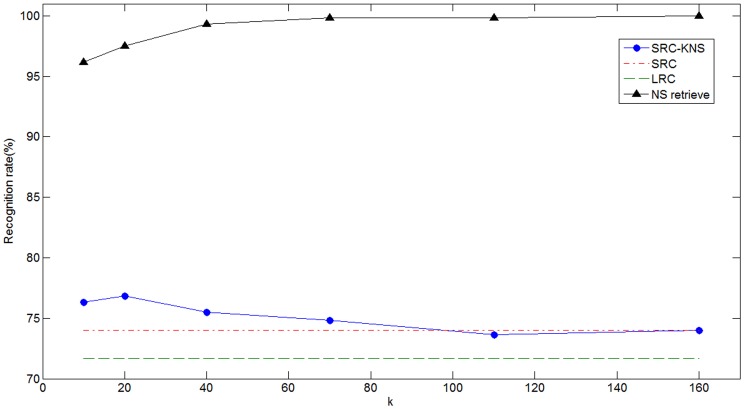
Recognition accuracy on the FERET database.

The FERET database is a very large publicly available database [31]. We used a subset consisting of 200 individuals in this experiment. For each subject, 4 images whose names are marked with two-character strings: “ba,” ”bj,” ” bk,” and ”be,” were used for training, and 3 others marked with two-character strings: “bf,” ”bd,” and ”bg,” were served as test samples. We downsampled the images to 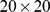. The results for SRC-KNS with 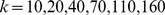 and SRC are listed in Table 3. We can see SRC-KNS (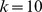) is around 5 times faster than SRC. Fig. 5 shows that SRC-KNS outperforms SRC in terms of recognition rate when 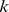 is less than 80. And they perform almost equally with the further increase of 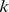. We can see retrieval accuracy of NS approaches 100% very quickly. Therefore, for the FERET database, NS shows great ability to retrieve the genuine class of a test sample, which insures the promising results of our methods.

**Table 3:** Comparison of CPU time on the FERET database.

Method	SRC-KNS						SRC
	*k* = 10	*k* = 20	*k* = 40	*k* = 70	*k* = 110	*k* = 160	
CPU time	0.784	0.838	0.968	1.156	1.516	2.096	4.016

Based on the above results on the three databases, each having significantly different number of subjects, we want to draw the following conclusions:

1. Compared with the original SRC, our method which uses 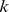 selected classes to perform SRC can achieve comparable recognition accuracy and even better in some occasions.
2. Using NS as the measurement of the relationship between a test sample and each class can achieve high accuracy of retrieving the genuine class of the test sample.
3. Without decreasing the recognition rate, the computational cost of SRC-KNS becomes lower as the 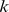 falls.
4. Although NS can find the 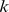 nearest subspaces of a test sample efficiently, recognition accuracy of NS-based classification method, namely LRC, is not comparable to that of SRC-KNS and SRC, which demonstrates the advantage of sparse representation-based method in terms of recognition rate.

### Recognize Images with Random Pixel Corruption

**Figure 6:**
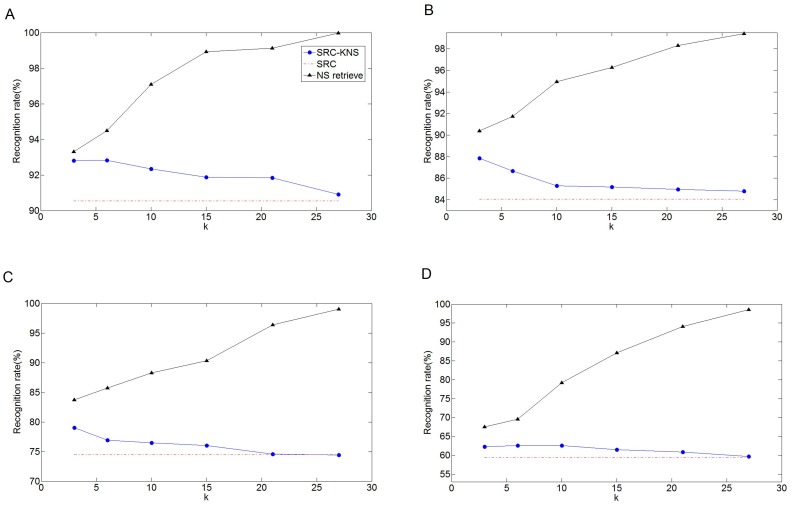
Recognition rates for test samples with different level of random corruption. (A) 10% random corruption (B) 20% random corruption. (C) 30% random corruption (D) 40% random corruption.

In this experiment, we tested our SRC-KNS under the condition that the images are contaminated by different levels of pixel corruption. Besides the corruption, the experimental protocol is consistent with the previous one used for the Extended Yale B database. Four different proportions of randomly chosen pixels of the test samples, i.e. 10%, 20%, 30%, and 40%, are corrupted by replacing their values with independent and identically distributed values from a uniform distribution which are in the range of 0 to 256. Table 4 shows that SRC-KNS (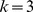) is 8 times faster than SRC. And the computation times for the four cases are consistent with each other approximately. Fig. 6 plots the recognition rate of SRC-KNS and SRC under four levels of corruption. We see that SRC-KNS outperforms SRC in most cases. And we can also see that it will benefit the recognition accuracy if using a smaller number of classes in this experiment. To explain this, we use the concept of the extent of sparsity concentration which is defined as the sparsity concentration index (SCI) [12]:
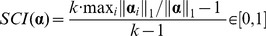
(11)


**Table 4:** Comparison of mean CPU time on the Extended Yale B database with different corruption levels.

Method		SRC-KNS						SRC
		*k* = 3	*k* = 6	*k* = 10	*k* = 15	*k* = 21	*k* = 27	
CPU time	10%	0.422	0.648	0.881	1.175	1.571	2.049	2.609
	20%	0.420	0.639	0.887	1.191	1.600	2.124	2.654
	30%	0.418	0.631	0.886	1.188	1.590	2.139	2.715
	40%	0.418	0.636	0.886	1.204	1.612	2.143	2.716

**Figure 7:**
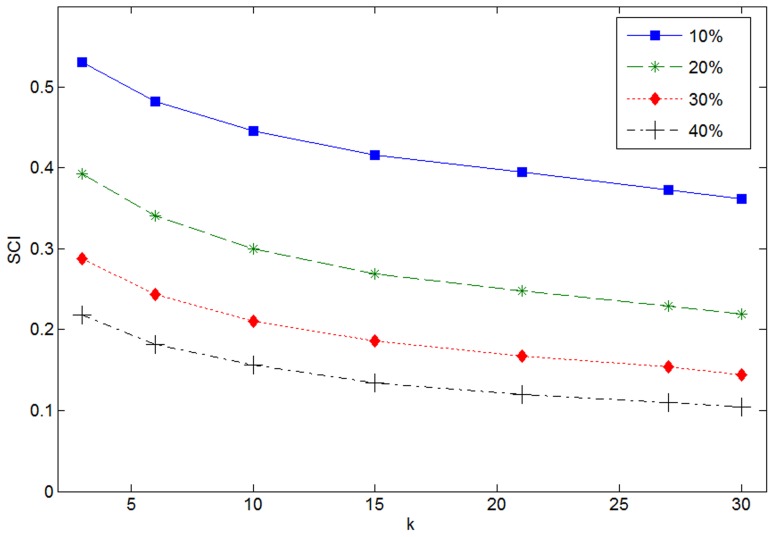
SCI of coding vectors under different levels of corruptions.

If a test sample is represented by training samples from a single class, then 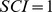. And the value of SCI declines along with the decrease of extent of concentration. Fig. 7 shows SCI curves are in function of the number of selected classes for different corruption levels. We see that to increase the corruption level makes representation less concentrative, or more classes make contributions to represent the test samples. Another observation is that, for a single SCI curve, the SCI value decreases with the increase of 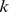. As we have known, 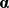 is more discriminative when its coefficients concentrate on a single class than that when the coefficients are spread evenly over all classes. Thus, based on the results, we argue when a portion of pixel of images are corrupted, smaller 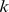 makes the coding coefficients of SRC-KNS more concentrative, which is benefit for recognition accuracy.

### Recognize Images with Contiguous Occlusion

**Figure 8:**
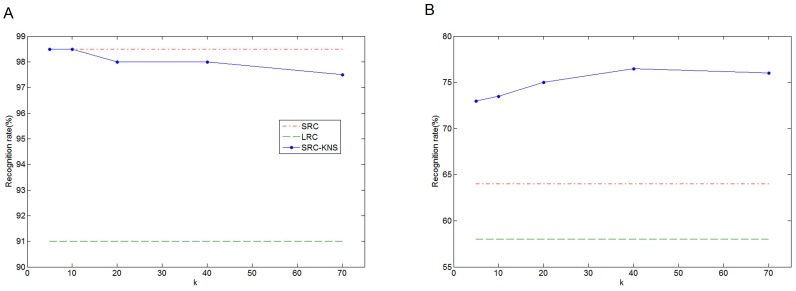
Recognition rates for occluded cases. (A) sunglass occlusion. (B) scarf occlusion.

In this experiment, we used images from AR database, consisting of two kinds of contiguous occlusion: the sunglasses and the scarf, to test the modular SRC-KNS. The same 100 subjects, as used in previous experiment, were used for evaluation. For each individual, 8 unoccluded images with varying facial expressions were served as training samples. The images with sunglasses and scarf were used for testing. Usually, higher resolution images are required to deal with occlusions [12], so that the images were downsampled to 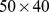. We compare our methods with the one proposed in [12] which is an extension of SRC method designed to cope with occlusions. In the extended SRC method, a unit matrix is added to 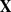, so we have a new dictionary 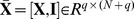. Since the occlusions contaminate only a portion of the test images, the solution for the extended SRC should be sparse as well (See [12] for further details). In modular SRC-KNS, we partitioned the images into 8 blocks (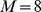) and sought 3 unoccluded blocks (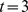). The results of computation time are listed in Table 5. Compared with the extended SRC, our method achieved 140 times speed-up (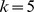), because the problem scales of these two methods are greatly different. That is, the dictionary used in the second phase of our method is 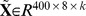
at most, whereas the extended SRC uses 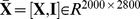. At the same time, recognition rates of the modular SRC-KNS, shown in Fig. 8, are comparable to that of the extended SRC when dealing with moderate occlusions, such as sunglasses. However, when identifying faces with severe occlusion, such as scarf, our method outperforms the extended SRC by 8% at least. We should mention that the LRC still yields the lowest recognition rates in both cases.

**Table 5:** CPU time (measured in second) for the occlusions on the AR database.

Method	SRC-KNS					Extended SRC
	*k* = 5	*k* = 10	*k* = 20	*k* = 40	*k* = 70	
CPU time for sunglasses	0.3650	0.4583	0.5761	0.7990	1.5355	52.630
CPU time for scarf	0.3573	0.4449	0.5650	0.7885	1.3930	55.837

### Conclusion

In this paper, we propose a novel method, called SRC-KNS, to perform sparse representation-based classification with high efficiency. The proposed method is evaluated using three publicly available databases. Although each database varies in the number of subjects, our method achieves 5 times speed-up at least compared to the original SRC when the selected nearest subspaces is less than 10% of total subjects. Meanwhile, the recognition accuracy of our method is comparable to SRC in general conditions. When identifying the test samples contaminated by some random pixel corruption, the experimental results reflects the fact that choosing less nearest subspaces helps to improve the recognition accuracy as well as speed up the algorithm. To cope with contingent occlusions, the modular SRC-KNS is proposed which measures the distance individually for each block. By using these distances, we introduce an indicator which is effective to identify 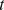 unoccluded blocks of the test sample. Since we use the unoccluded blocks as feature, the 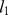-minimization problem solved in our modular SRC-KNS has a much smaller size than the one in the extended SRC. When coping with the severely occluded images, our method outperforms the extended SRC on both recognition rate and computational efficiency.

The SRC is a state-of-art method that performs robust face recognition very well. However, when there is a large quantity of the training samples, SRC is very time-consuming. We use NS method to very effectively and efficiently select a small subset from the entire training set that is very likely to include the class of the test sample. After the sample selection process, SRC also works well but only needs to solve a smaller 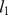-minimization problem. Our study suggests the proposed scheme of conducting SRC has no recognition rate impairments, and sometimes enhances the recognition accuracy, especially for recognizing corrupted samples. We hope the proposed methods can push forth the implementation of SRC-based technique in the realistic face recognition applications in future.
